# A deazariboflavin chromophore kinetically stabilizes reduced FAD state in a bifunctional cryptochrome

**DOI:** 10.1038/s41598-023-43930-0

**Published:** 2023-10-04

**Authors:** Yuhei Hosokawa, Hiroyoshi Morita, Mai Nakamura, Junpei Yamamoto

**Affiliations:** Graduate School of Engineering Science, Osaka University, 1-3 Machikaneyama, Toyonaka, Osaka 560-8531 Japan

**Keywords:** Kinetics, Photobiology, Biophysical chemistry

## Abstract

An animal-like cryptochrome derived from *Chlamydomonas reinhardtii* (*Cr*aCRY) is a bifunctional flavoenzyme harboring flavin adenine dinucleotide (FAD) as a photoreceptive/catalytic center and functions both in the regulation of gene transcription and the repair of UV-induced DNA lesions in a light-dependent manner, using different FAD redox states. To address how *Cr*aCRY stabilizes the physiologically relevant redox state of FAD, we investigated the thermodynamic and kinetic stability of the two-electron reduced anionic FAD state (FADH^−^) in *Cr*aCRY and related (6–4) photolyases. The thermodynamic stability of FADH^−^ remained almost the same compared to that of all tested proteins. However, the kinetic stability of FADH^−^ varied remarkably depending on the local structure of the secondary pocket, where an auxiliary chromophore, 8-hydroxy-7,8-didemethyl-5-deazariboflavin (8-HDF), can be accommodated. The observed effect of 8-HDF uptake on the enhancement of the kinetic stability of FADH^−^ suggests an essential role of 8-HDF in the bifunctionality of *Cr*aCRY.

## Introduction

While sunlight is indispensable for the evolution of life on Earth, solar energy in the UV-B and -C ranges (100–315 nm) exerts a harmful effect on DNA by altering the chemical structures of adjacent pyrimidine bases into lesions represented by cyclobutene pyrimidine dimers (CPDs) and/or pyrimidine(6–4)pyrimidone photoproducts ((6–4)PPs), which potentially cause mutagenesis and carcinogenesis^1,2^. To maintain genetic integrity, many organisms possess photolyases (PLs), flavoproteins able to repair the DNA lesions to their original structures using blue light^3^. Despite classification of PLs into CPD PLs and (6–4) PLs based on their substrates, bond rearrangement via photoinduced electron transfer from a flavin adenine dinucleotide (FAD) cofactor to the substrates is a common key reaction mediated by PLs^4,5^. While FAD in PLs can be observed in three redox states, namely, oxidized (FAD_ox_), one-electron reduced neutral (FADH^·^), or two-electron reduced anionic (FADH^−^) forms, only the FADH^−^ state can be used for the repair reaction. Because of the prerequisite of the FADH^−^ state for their activity, PLs developed a unique process in which FAD is reduced in a light-dependent manner, called photoreduction^6^. In addition to the FAD cofactor, an auxiliary chromophore can be found in some PLs and is known to absorb photon energy, transfer it to FAD through the Förster resonance energy transfer (FRET) mechanism^7^, and assist with the photoexcitation of FADH^−^ for the photorepair reaction^8^. So far, 8-hydroxy-7,8-didemethyl-5-deazariboflavin (8-HDF) has been identified as an antenna chromophore in some (6–4) PLs^9–12^.

Cryptochromes (CRYs) are phylogenetically relevant to PLs with similar structural architectures but are involved in various biological events apart from DNA repair, such as circadian rhythm regulation^13^. Therefore, PLs and CRYs form a flavoprotein family called the **P**hotolyase/**C**ryptochrome **S**uper**f**amily (PCSf). Several types of CRYs from plants and animals have been identified as photoreceptive CRYs bearing FAD. For example, plant CRYs are representative photoreceptors that transmit light signals to the blue light signaling pathways, which regulate photomorphogenic behaviors^14–16^ and circadian rhythm entrainment^17^. In addition to plant CRYs that have evolved from CPD PLs, *Drosophila*-type CRYs (dCRYs), which are evolutionarily derived from (6–4) PLs, have also been characterized as photoreceptors that primarily function in synchronization of the circadian rhythm with sunlight^18^. Unlike PLs functioning in the FADH^−^ state, these photoreceptive CRYs appear to interact with other partner proteins in not FADH^−^ but the one-electron reduced form (FADH^·^ for plant CRYs^19^ or a one-electron reduced anionic state (FAD^·−^) for dCRYs^20^). Hence, understanding the mechanism by which PCSf proteins with similar frameworks differently stabilize the FAD redox states required for their biological actions provides a fundamental clue for the origin of their functional diversity.

In previous studies^21–25^, the residue next to the N5 atom of isoalloxazine in FAD was found to be related to the functional redox states of FAD in PCSf proteins. An Asp residue in plant CRYs enables rapid generation of its active FADH^·^ state by protonating FAD^·−22^. In contrast, Cys residues in dCRYs reportedly inhibit protonation, yielding an active FAD^·−^ state^23^. In both CPD and (6–4) PLs, an Asn residue is exclusively conserved at the position^6^. In particular, the Asn residue in CPD PLs stabilizes the FADH^·^ state in both kinetic and thermodynamic manner for the repair reaction performed with the FADH^·^/FADH^–^ redox pair^24^. A time-resolved crystallographic study of FAD photoreduction in a PCSf protein captured the transient motion of the Asn residue to stabilize the FADH^·^ state, thereby acting as a redox sensor triad together with the proximal Arg–Asp salt bridge^25^. These examples indicate that the N5-proximal Asp, Asn, and Cys residues exclusively govern the functional FAD state in PCSf proteins. However, the recent discovery of animal-like CRY from *Chlamydomonas reinhardtii* (*Cr*aCRY) challenges the paradigm. *Cr*aCRY is found to exhibit not only photoreceptive CRY functions regulating the transcription of genes^26^ but also the ability to repair (6–4)PPs using FADH^−^^27^. Interestingly, *Cr*aCRY has an Asn residue proximal to the N5 atom of isoalloxazine of FAD and adopts FAD_ox_, FADH^·^, and FADH^−^ states^28^ as well as (6–4) PLs do. Therefore, it is challenging to determine how *Cr*aCRY sharing a similar architecture and redox chemistry of FAD with those of (6–4) PLs executes additional CRY functions.

In this study, we shed light on the molecular origin of the unique functionality of *Cr*aCRY by determining the thermodynamic stability of the FADH^−^ state and its kinetic stability against the reoxidation reaction in *Cr*aCRY and its evolutionarily related (6–4) PLs. Consistent with the conservation of residues around FAD, the thermodynamic stability did not show a significant difference between them. However, the kinetic stability of FADH^−^ in the tested proteins varied remarkably depending on the local structure of the secondary pocket, where natural antenna chromophores, such as 8-HDF, can be accommodated. Based on these results, we propose that the bifunctionality of *Cr*aCRY is likely regulated by the presence or absence of 8-HDF.

## Results

### Evaluation of thermodynamic stability of FADH^−^ in (6–4) PLs

**Figure 1:**
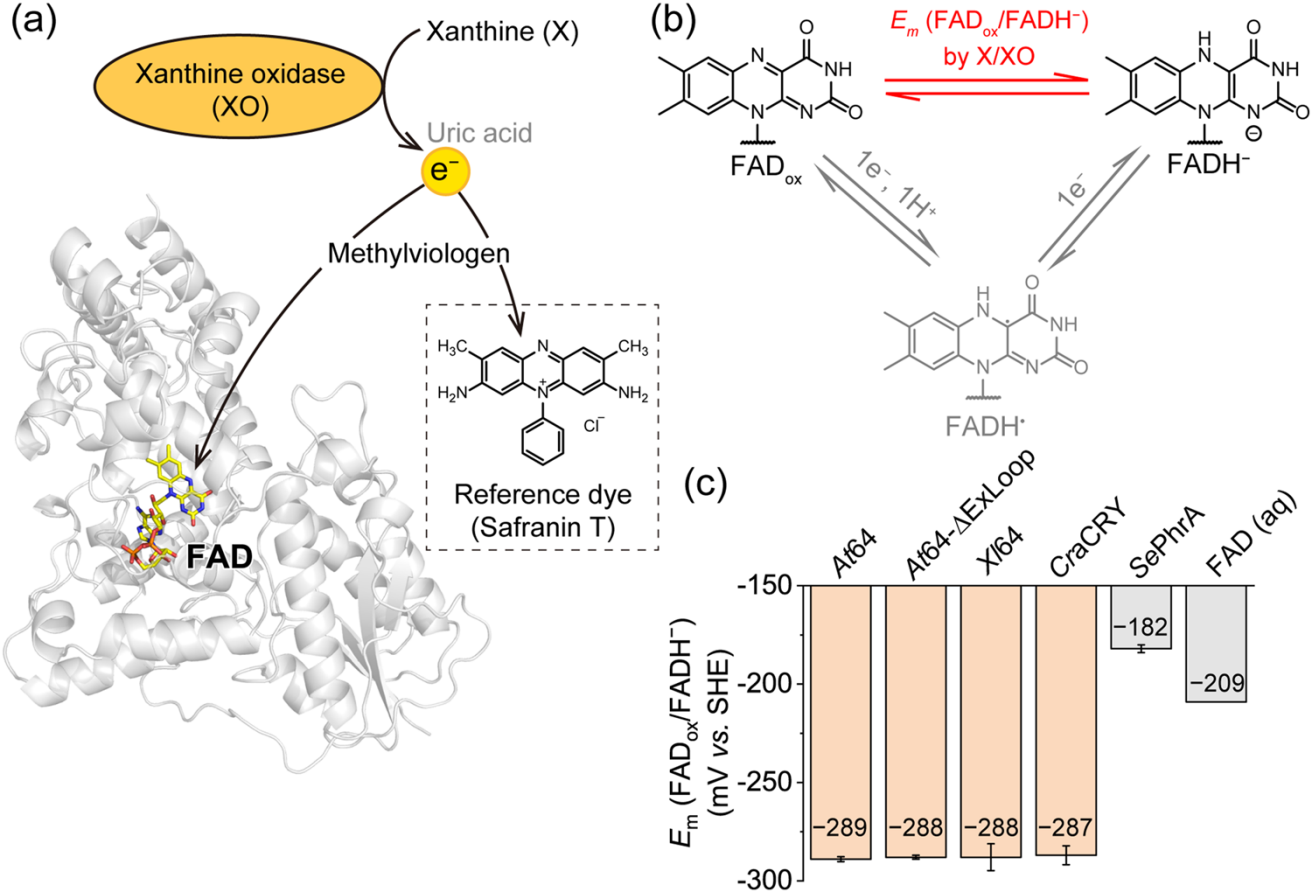
Evaluation of thermodynamic stabilities of FADH^−^ in (6–4)PP-repairing proteins by the xanthine/xanthine oxidase (X/XO) method. (**a**) The oxidation of X to uric acid by XO supplies electrons to FAD and a reference dye Safranin T via a redox mediator methylviologen. The structural model was taken from the reported crystal structure of *At*64 (PDB: 3FY4)^32^. (**b**) The X/XO reaction reduced fully oxidized FAD_ox_ to two-electron reduced anionic FADH^−^ without accumulating one-electron reduced FADH^·^. (**c**) Midpoint potentials of the FAD_ox_/FADH^−^ redox pair in (6–4)PP-repairing proteins were ~  − 290 mV *vs.* SHE. The value was considerably lower than those reported for CPD PL derived from *Synechococcus elongatus* (*Se*PhrA), formerly called *Anacystis nidulans* CPD PL^11,24^ and for the solution FAD (aq) state^33^. Error bars for our samples reflect the standard deviation for *n* = 3.

We first evaluated the thermodynamic stability of FADH^−^ in (6–4) PLs from *Arabidopsis thaliana* and *Xenopus laevis* (*At*64 and *Xl*64) and *Cr*aCRY using a spectroscopic electrochemical analysis called the xanthine/xanthine oxidase (X/XO) method^29^. In the well-established technique to evaluate the midpoint potentials of flavin derivatives in flavoproteins, electrons released from the oxidation of X by XO simultaneously reduced FAD and a reference dye in the presence of the redox mediator methylviologen (Fig. 1a). After testing various reference dyes, we found that Safranin T dye was reduced simultaneously with FAD in *At*64, *Xl*64, and *Cr*aCRY. During the reduction by X/XO, no accumulation of the one-electron reduced FADH^·^ state was observed, as confirmed by probing the absorption unique to FADH^·^ at wavelengths from 500 to 700 nm (Supplementary Fig. 1). The findings indicated that the two-electron reduction of FAD_ox_ into FADH^−^ occurred in the system (Fig. 1b). Accordingly, the synchronous decreases in the absorption characteristic to the oxidized states of FAD and Safranin T (A_450_ for FAD_ox_, A_510_ for Safranin T in Supplementary Figs. 2–4) were analyzed using Eq. 1, as described in *Methods*.

1 $$\begin{array}{*{20}c} {12.8\ln \frac{{\left[ {{\text{D}}_{{{\text{ox}}}} } \right]_{t} }}{{\left[ {{\text{D}}_{{{\text{red}}}} } \right]_{t} }} = 12.8\ln \frac{{\left[ {{\text{FAD}}_{{{\text{ox}}}} } \right]_{t} }}{{\left[ {{\text{FADH}}^{ - } } \right]_{t} }} + E_{{{\text{m}},{\text{FAD}}}} - E_{{{\text{m}},{\text{D}}}} } \\ \end{array}$$

In Eq. 1, the concentration ratios between the oxidized and two-electron reduced states of FAD and Safranin T ([FAD_ox_]_*t*_ and [FADH^−^]_*t*_ for FAD, [D_ox_]_*t*_ and [D_red_]_*t*_ for the dye, where* t* indicates the time at which the absorbance is measured) are related to the midpoint potentials of FAD and Safranin T (*E*_m,FAD_ and *E*_m,D_, respectively; *E*_m,D_ =  − 289 mV *vs.* SHE (Standard Hydrogen Electrode) for Safranin T). The plots of the data (12.8 ln ([FAD_ox_]_*t*_/[FADH^−^]_*t*_) *vs.* 12.8 ln ([D_ox_]_*t*_/[D_red_]_*t*_)) were reproducibly fitted to the equation, yielding a linear correlation with a slope of ~ 1 (Supplementary Figs. 2–4). Based on the fitting of the data with the equation, we concluded that the *E*_m,FAD_ values of the tested proteins (*Xl*64, *At*64, and *Cr*aCRY) were identical, to be − 290 mV *vs.* SHE (Fig. 1c). Surprisingly, the value was considerably lower than that for CPD PLs (− 182 mV *vs.* SHE) reported by Damiani et al.^24^, indicating that the FADH^−^ state in (6–4) PLs was thermodynamically destabilized compared to that in CPD PLs even though both (6–4) PLs and CPD PLs commonly utilize FADH^−^ as the active redox form for the DNA repair. This finding is consistent with previous reports on the FADH^−^ stability of *Escherichia coli* CPD PL and *Xl*64 in bacterial cells^30,31^. The difference in the thermodynamic stability of the FADH^−^ state could be attributed to the structural variations around FAD between (6–4) PLs and CPD PLs (Supplementary Fig. 5).

### Evaluation of kinetic stability of FADH^−^ in (6–4) PLs

**Figure 2:**
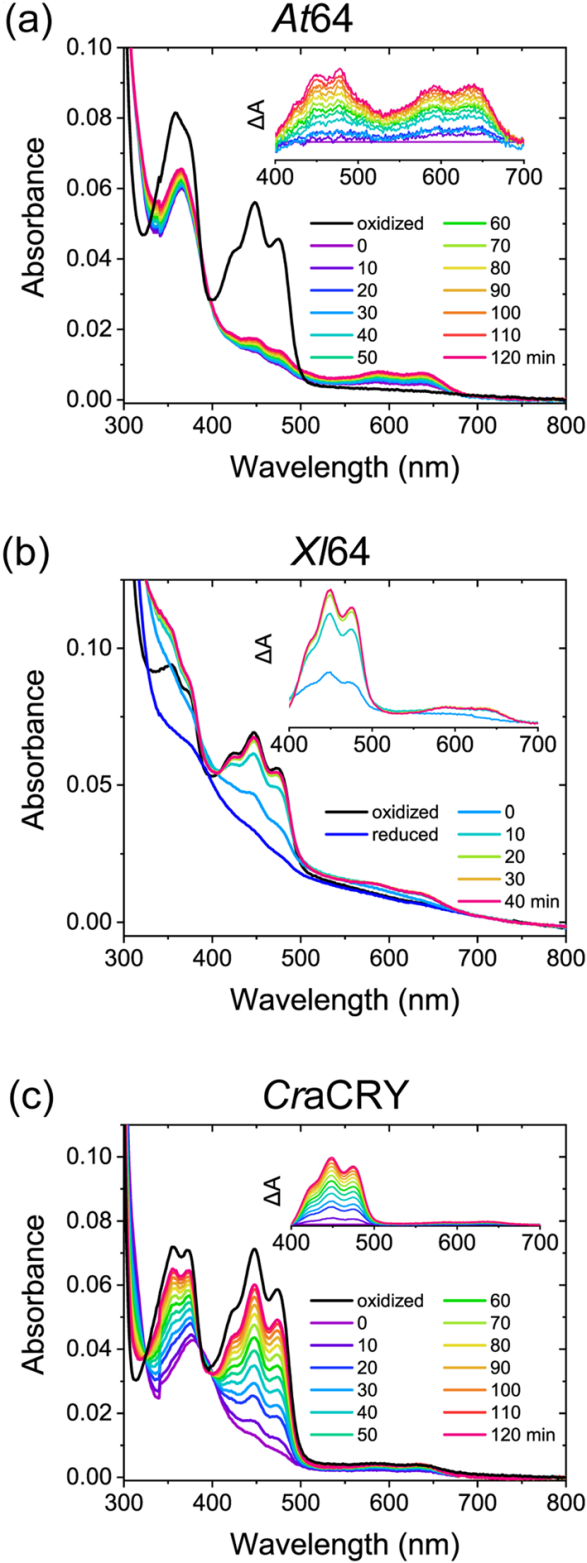
Spectral changes associated with oxidation of the photoreduced (**a**) *At*64, (**b**) *Xl*64, and (**c**) *Cr*aCRY samples. The spectrum was recorded every 10 min after starting the oxidation. Insets for (**a**) and (**c**) show the difference spectra in which each spectrum was subtracted by the spectrum at 0 min recorded at wavelengths from 400 to 700 nm. In panel (**b**), light scattering due to protein aggregation can be seen particularly in the wavelength range of 300–400 nm. This effect may be attributed to *Xl*64 being dissolved in a buffer optimized for *At*64 and *Cr*aCRY, to maintain consistent buffer conditions across all samples. In our previous experiment using a buffer optimized for *Xl*64, a similar kinetic behavior to that presented here was observed without the scattering^31^. This suggests that the fast kinetics of *Xl*64 does not result from unstable protein folding. An inset for (**b**) shows the difference spectra in which each spectrum was subtracted by the spectrum of the reduced state, indicating that the considerable amount of FADH^−^ was already reoxidized to FAD_ox_ immediately after triggering the reaction. The difference spectra, which exhibit a typical change upon reoxidation, also support that the protein quality is sufficient for characterizing the kinetic property of *Xl*64.

While our midpoint potential measurements showed no differences in the *E*_m,FAD_ values among (6–4)PP-repairing proteins derived from different organisms, a previous study reported that FADH^−^ produced by photoreduction was reoxidized to FAD_ox_ much faster in *Xl*64 than in *At*64^31^. This implies that (6–4) PLs would exhibit a variety of kinetic stabilities of FADH^−^ in response to molecular oxygen depending on the species, whereas the kinetic parameters of the reoxidation in (6–4) PLs have not been reported. To evaluate the kinetics, we first photoreduced FAD to FADH^−^ in a protein under anaerobic conditions and then exposed the sample to ambient air to induce reoxidation of FADH^−^ to FAD_ox_ and/or FADH^·^. The UV/vis absorption spectrum was recorded at 15 °C every 10 min for 2 h to follow the reoxidation (Fig. 2). In all cases, the recovery of FAD_ox_ (absorption band of 400–500 nm) and some accumulation of FADH^·^ (broad absorption band of 550–700 nm) were observed, but their timescales were completely varied. For *Xl*64, the spectral feature of FAD_ox_ was mostly recovered in the first 10 min of reoxidation (Fig. 2b). Although the reoxidation in *Xl*64 was too fast to be analyzed using our experimental setup, the reoxidation in *At*64 and *Cr*aCRY occurred over a measurement time of 2 h (Fig. 2a and c).

The reoxidation of FADH^−^ in PCSf proteins involves two pathways^24,34^ (Supplementary Scheme S1): (1) a sequential one-electron transfer pathway, which consists of two steps, namely, the oxidation to FADH^·^ with a rate constant *k*_1_ and the oxidation of FADH^·^ to FAD_ox_ with a rate constant *k*_2_ and (2) a direct two-electron oxidation pathway to FAD_ox_ with a rate constant *k*_3_. According to the scheme, changes in the concentration of FADH^·^ upon reoxidation (Δ[FADH^·^]) should be simulated using Eq. 2.

2 $$\begin{aligned} \Delta \left[ {{\text{FADH}}^{ \cdot } } \right] = & \left[ {{\text{FADH}}^{ - } } \right]_{0} \frac{{k_{1} }}{{k_{2} - k_{1} - k_{3} }}\left[ {exp\left\{ { - \left( {k_{1} + k_{3} } \right)t} \right\} - exp\left( { - k_{2} t} \right)} \right] \\ & + \left[ {{\text{FADH}}^{ \cdot } } \right]_{0} \left\{ {exp\left( { - k_{2} t} \right) - 1} \right\} \\ \end{aligned}$$

However, Δ[FADH^·^] in the tested proteins was fitted with single-component exponential functions rather than two-component exponential functions (Supplementary Fig. 6). One possible explanation for this result is that the *k*_2_ value is much smaller than *k*_1_ and/or *k*_3_ (*k*_2_ <  < *k*_1_ + *k*_3_). In this case, Δ[FADH^·^] can be simulated using Eq. 3.

3 $$\begin{array}{*{20}c} {\Delta \left[ {{\text{FADH}}^{ \cdot } } \right] = - \left[ {{\text{FADH}}^{ - } } \right]_{0} \frac{{k_{1} }}{{k_{1} + k_{3} }}\left[ {exp\left\{ { - \left( {k_{1} + k_{3} } \right)t} \right\} - 1} \right]} \\ \end{array}$$

When Eq. 3 holds, the oxidation of FADH^·^ to FAD_ox_ would no longer occur during our measurement time. Indeed, the slowly increasing absorption in the wavelength range of 550–700 nm in the spectra ensured that the amount of FADH^·^ produced by reoxidation was limited so that the contribution of the reoxidation of FADH^·^ to FAD_ox_ would be negligible. Therefore, we analyzed the rate constants *k*_1_ and *k*_3_ that control the reoxidation of FADH^−^ to FADH^·^ and FAD_ox_, respectively, based on the *k*_2_ <  < *k*_1_ + *k*_3_ approximation (Table 1).

**Table 1:** Reoxidation kinetics of FADH^−^ in (6–4)PP-repairing proteins.

	*k*_1_ (× 10^−3^ min^−1^)	*k*_3_ (× 10^−3^ min^−1^)	*k*_1_ + *k*_3_ (× 10^−3^ min^−1^)	*F*_reox1_ = *k*_1_/(*k*_1_ + *k*_3_)	*F*_reox2_ = *k*_3_/(*k*_1_ + *k*_3_)
*At*64	1.27	0.422	1.69 ± 0.04	0.75	0.25
*At*64-ΔExLoop	1.29	2.67	3.96 ± 0.06	0.33	0.67
*Xl*64^*a*^	N.D	N.D	N.D	N.D	N.D
*Xl*64-HDF	2.79	1.85	4.64 ± 1.28	0.60	0.40
*Cr*aCRY	0.559	9.33	9.89 ± 0.75	0.057	0.94
*Cr*aCRY-HDF	1.42	0.936	2.35 ± 0.20	0.60	0.40

^*a*^For *Xl*64, the reoxidation of FADH^−^ was too fast to estimate the rate constants with our experimental set-up.

In agreement with the differences in time-dependent spectral changes between *At*64 and *Cr*aCRY (Fig. 2a and c), the rate constants calculated for *At*64 differed from those for *Cr*aCRY. According to the *k*_1_ + *k*_3_ value, which reflects the total reoxidation kinetics of FADH^−^ to FADH^·^ and FAD_ox_, reoxidation of FADH^−^ was 5.9-fold faster in *Cr*aCRY than in *At*64. Based on the obtained spectra and rate constants, we simulated the time-dependent changes in the concentration of FAD in each redox state for *At*64 and *Cr*aCRY (Supplementary Fig. 6a and c) and found that the stability of FADH^−^ in *At*64 was much higher than that in *Cr*aCRY.

To quantify the contribution of the one- and two-electron transfer pathways to FADH^–^ reoxidation, fractions of *k*_1_ and *k*_3_ in the total reoxidation *k*_1_ + *k*_3_ value (*F*_reox1_ and *F*_reox2_, respectively) were introduced. Comparison of these values show a different profile between *At*64 and *Cr*aCRY (Table 1), where *F*_reox2_ for *At*64 was 25% while that for *Cr*aCRY was 94%. This observation clearly suggests that *Cr*aCRY is more subjected to the two-electron oxidation of FADH^−^ than *At*64. A previous study on plant CRY suggested that two-electron reoxidation involves two successive oxidation steps^34^: oxidation of FADH^−^ to FADH^·^ by the reduction of O_2_ to the O_2_^·−^ anion radical, and subsequent oxidation of FADH^·^ by the HO_2_^·^ hydroperoxyl radical, the protonated form of O_2_^·−^. Considering that molecular oxygen must be present in close proximity to FAD during the FAD reoxidation process, the surroundings of FAD in *Cr*aCRY may be optimized for immediate protonation of O_2_^·−^, enhancing direct reoxidation of FADH^−^ to FAD_ox_. Our kinetic analyses not only showed that (6–4)PP-repairing proteins exhibited a variety of kinetic stabilities of FADH^−^ against molecular oxygen but also suggested a diversity of mechanisms that regulate the reoxidation of FADH^−^.

### The kinetic stability of FADH^−^ in (6–4) PLs regulated by the secondary pocket

**Figure 3:**
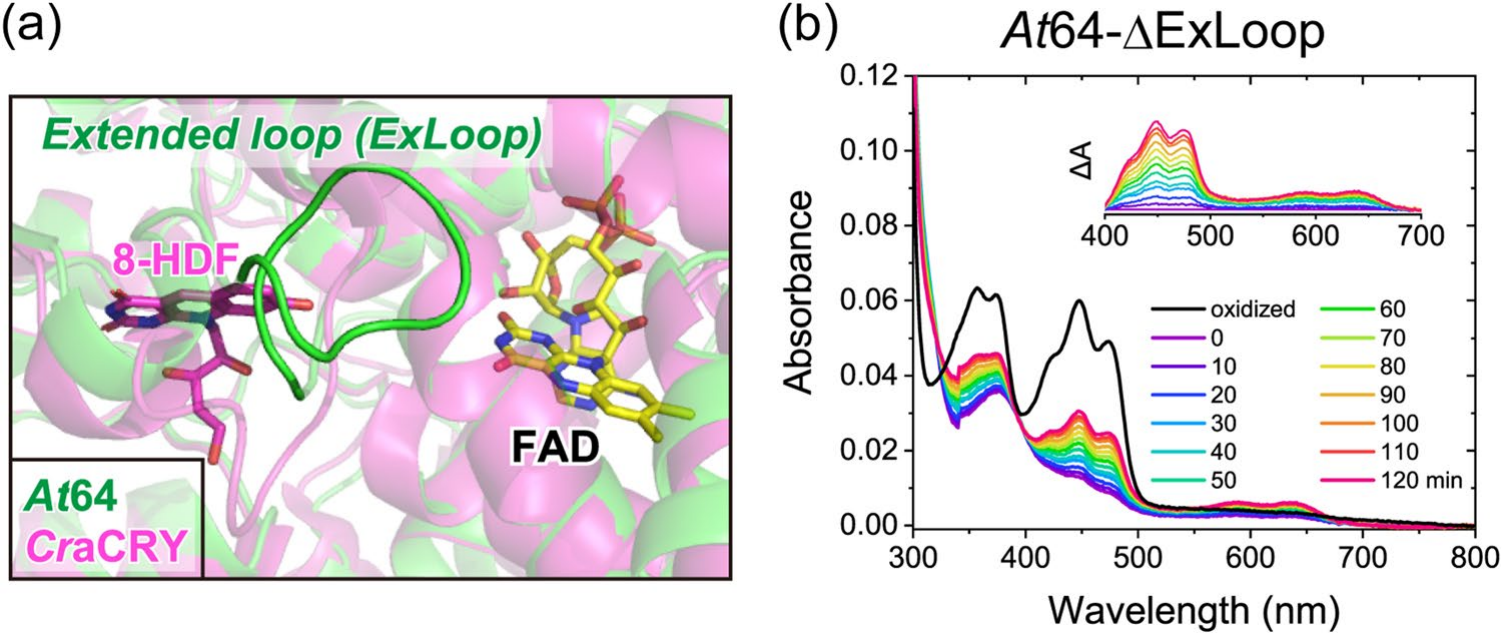
Effects of a plant-specific extended loop on the kinetic stability of FADH^−^. (**a**) Structural comparison of *At*64 (PDB: 3FY4^32^, in green) and *Cr*aCRY in complex with 8-HDF (PDB: 6FN2^27^, in magenta) shows the presence of the extended loop around the secondary pocket in *At*64. (**b**) Spectral changes associated with oxidation of the photoreduced *At*64-ΔExLoop sample were recorded every 10 min. An inset shows the difference spectra in which each spectrum was subtracted by the spectrum at 0 min recorded at wavelengths from 400 to 700 nm.

To investigate the origin of the observed differences in the kinetic stability of FADH^−^ among the (6–4)PP-repairing proteins, we compared their three-dimensional structures. As inferred from the shared redox potential of FAD, most of the residues within 5 Å of FAD were conserved (90.6% amino acid sequence identity between *At*64 and *Cr*aCRY; 81.3% amino acid sequence identity between *At*64 and *Xl*64; Supplementary Fig. 7). Apart from the conserved FAD binding site, we focused on the secondary pocket, which potentially accommodates a secondary chromophore in many PCSf proteins. Because the pocket is known to be a mutational hotspot co-evolving with protein functions in animal CRYs^35^, the structural diversity in the secondary pocket among (6–4)PP-repairing proteins may be related to the kinetic variations of the reoxidation process. Regarding (6–4)PP-repairing proteins, *Xl*64 and *Cr*aCRY can harbor 8-hydroxy-7,8-didemethyl-5-deazariboflavin (8-HDF) as the antenna chromophore, transferring its absorbed energy to FAD^10,27^. However, a previous study^12^ suggested that *At*64 would completely lose its ability to bind 8-HDF, presumably because of the exclusive presence of an extended loop near the secondary pocket (Fig. 3a). As the presence of the extended loop in *At*64 would also inhibit O_2_ coming proximal to the FAD chromophore, we evaluated the effect of the extended loop on the thermodynamic and kinetic stability of FADH^−^ in *At*64.

The mutant lacking the extended loop (*At*64-ΔExLoop) exhibited a spectral feature shared with the wild type *At*64, indicating that the whole structure remained essentially unchanged. As the deletion of the extended loop did not affect the FAD binding site, the *E*_m,FAD_ value of *At*64-ΔExLoop remained unchanged (− 288 mV *vs.* SHE) (Fig. 1c and Supplementary Fig. 8). However, the spectral development upon reoxidation in *At*64-ΔExLoop was significantly different from that in *At*64 (Fig. 2a *vs.* Fig. 3b). The whole decay kinetics of FADH^−^ (*k*_1_ + *k*_3_) in *At*64-ΔExLoop was 2.3-fold accelerated by the deletion (Table 1), indicating that the kinetic stability of FADH^−^ was significantly lowered by the deletion of the extended loop (Supplementary Fig. 6). Because *At*64-ΔExLoop has a similar *k*_1_ value to that of *At*64 but a 6.3-fold larger *k*_3_ value than that of *At*64, truncation of the extended loop would enhance the two-electron oxidation of FADH^−^. Indeed, *F*_reox2_ for *At*64 and *At*64-ΔExLoop were 25% and 67%, respectively. This phenomenon may correspond to the observation that *Cr*aCRY, which does not have an extended loop, favors two-electron oxidation over one-electron oxidation.

### Effects of 8-HDF on the kinetic stability of FADH^−^ in *Xl*64 and *Cr*aCRY

Considering that the extended loop near the secondary pocket affects the reoxidation kinetics of FADH^−^ in *At*64, we evaluated the effects of 8-HDF binding on the kinetic stability of FADH^−^ in *Xl*64 and *Cr*aCRY. Chemically synthesized 8-HDF was bound to *Xl*64 and *Cr*aCRY as previously described^12^. *Xl*64 and *Cr*aCRY complexed with 8-HDF (*Xl*64-HDF and *Cr*aCRY-HDF) exhibited absorption maxima at 441 and 448 nm, respectively. The extent of the red-shift as compared to the absorption maximum of 8-HDF in solution (422 nm at pH 7) was coincident with those reported in previous studies^12,36^.

**Figure 4:**
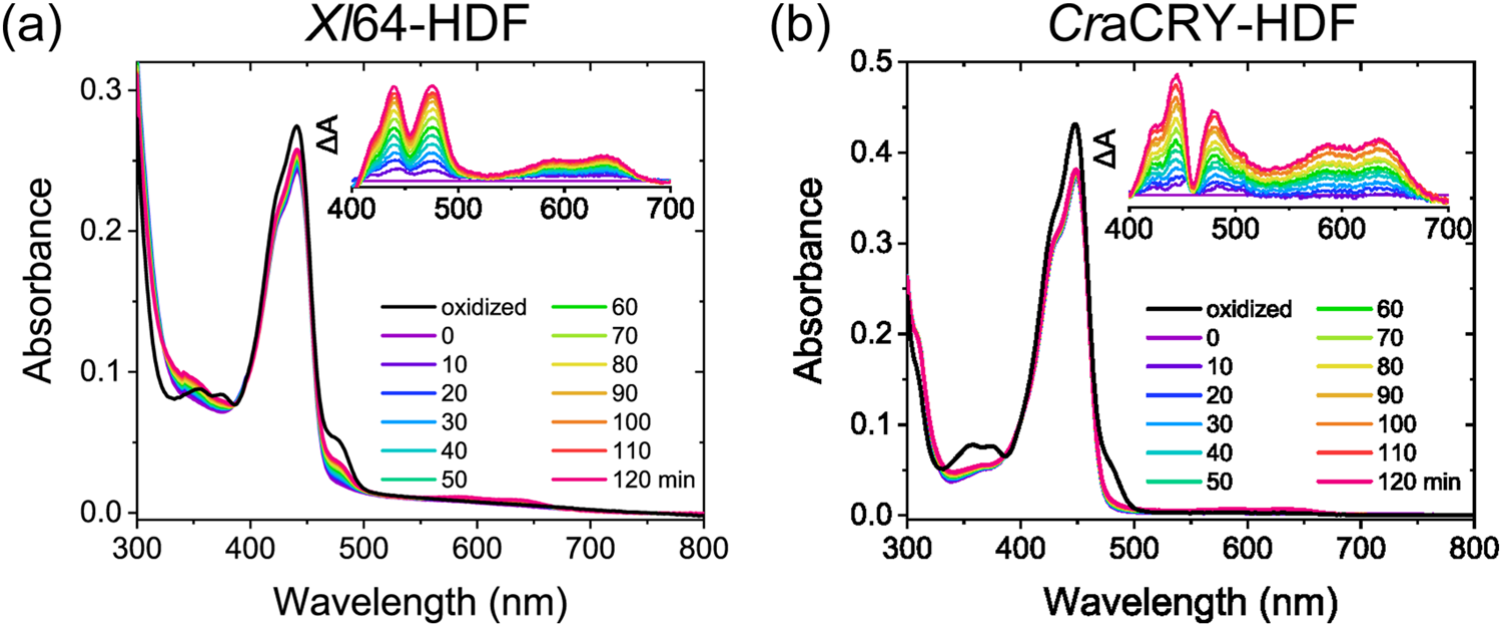
Spectral changes associated with oxidation of the photoreduced (**a**) *Xl*64-HDF and (**b**) *Cr*aCRY-HDF samples. The spectrum was recorded every 10 min after starting the oxidation. Insets represent the difference spectra in which each spectrum was subtracted by the spectrum at 0 min recorded at wavelengths from 400 to 700 nm.

Contrary to the very fast reoxidation process for *Xl*64, the reoxidation of FADH^−^ in *Xl*64-HDF occurred in a measurable time window of over 2 h (Fig. 4a). This clearly suggests that the occupation of the secondary pocket by 8-HDF decelerated the reoxidation of FADH^−^ and kinetically stabilized FADH^−^ against molecular oxygen in *Xl*64. Compared with the typical difference spectra observed for the apo-proteins (Figs. 2 and 3), the difference spectra for *Xl*64-HDF upon reoxidation showed a valley at 455 nm. This suggests that some spectral characteristics of 8-HDF would change upon reoxidation. The previous study^12^ reported that the 8-hydroxy group in 8-HDF exhibits a p*K*_a_ value of 6.1 and that Coulombic interactions between the phenoxide group in 8-HDF and positively charged residues in *Xl*64 are required for 8-HDF uptake by *Xl*64. Therefore, the spectral changes associated with 8-HDF upon the reoxidation of FADH^−^ are assumed to be attributed to the structural perturbation of the protein matrix around 8-HDF, not to changes in the protonation state of 8-HDF. The rate constants *k*_1_ and *k*_3_ for *Xl*64-HDF were calculated in the same manner as for the proteins without the antenna chromophore (Table 1). To verify whether the valley interferes with the kinetic analysis, we globally fitted the absorption changes in the wavelength range of 420–500 nm, which can be sensitive to reoxidation, using exponential functions with the obtained *k*_1_ + *k*_3_ rate constant. The absorption changes in that range were successfully fitted with a coefficient of determination of 0.998 (Supplementary Fig. 9a). Furthermore, the amplitude spectra, which correspond to the spectral component involved in reoxidation, were superimposable to the difference spectra upon reoxidation (Supplementary Fig. 9b). These results indicate that the valley observed in the difference spectra does not have a significant impact on our analysis. The obtained rate constants accurately simulated the time development of the concentrations of each redox state of FAD (Supplementary Fig. 6c). The results also showed that approximately half of FADH^−^ was not subjected to reoxidation within 2 h in *Xl*64-HDF, whereas the majority of FADH^−^ in *Xl*64 was immediately converted to FAD_ox_.

The difference spectra for *Cr*aCRY-HDF upon reoxidation exhibited a trend similar to that for *Xl*64-HDF, with a deeper valley at 461 nm (Fig. 4b) than that observed for *Xl*64-HDF at 455 nm. After calculating the kinetic parameters for *Cr*aCRY-HDF, we validated our analysis of the kinetic parameters for *Cr*aCRY-HDF, as well as the analysis for *Xl*64-HDF (Supplementary Fig. 9c and d). Although FADH^−^ in *Cr*aCRY was almost completely reoxidized to FAD_ox_ after 2 h, the spectrum of *Cr*aCRY-HDF after 2 h of reoxidation was far from that of the initial oxidized state (Fig. 2c *vs.* Fig.  4b). In agreement with this observation, the total reoxidation kinetic parameter *k*_1_ + *k*_3_ for *Cr*aCRY-HDF was 4.2-fold lower than that for *Cr*aCRY, indicating that the reoxidation of FADH^−^ in *Cr*aCRY-HDF was much slower than that in *Cr*aCRY (Table 1, Supplementary Fig. 6e). Interestingly, *F*_reox1_ was going up from 5.7 to 60% upon the accommodation of 8-HDF in *Cr*aCRY, and the preference for the reoxidation process was completely inverted, suggesting that the presence of 8-HDF would increase the population of FADH^·^. A similar preference for one-electron oxidation for *Cr*aCRY-HDF was also observed for *Xl*64-HDF, as shown by a similar *F*_reox1_ value of ~ 0.60 (Table 1). Given the observation that (i) two-electron oxidation was inhibited in *Cr*aCRY by the occupation of the secondary pocket by 8-HDF and (ii) two-electron oxidation was accelerated in *At*64 by the removal of the extended loop near the pocket, the accessibility of molecular oxygen to the secondary pocket is considerably related to the reoxidation pathways of FADH^−^ in (6–4)PP-repairing proteins. Hence, we conclude that the structure around the secondary pocket, including the presence and absence of 8-HDF, remarkably affects not only the lifetime of FADH^−^ against reoxidation but also the reoxidation mechanism.

## Discussion

We investigated the thermodynamic and kinetic stabilities of the FADH^−^ state in (6–4)PP-repairing proteins to gain new insights into how (6–4) PLs and *Cr*aCRY regulate the redox chemistry of FAD for their activity. The thermodynamic stability of FADH^−^ was found to be commonly lower in the proteins than in the solution state (*E*_m,FAD_ of ~  − 290 mV *vs.* SHE for the proteins; *E*_m,FAD_ of ~  − 209 mV *vs.* SHE for the solution state^33^). In contrast to their shared thermodynamic stability, they showed a variety of kinetic stabilities of FADH^−^ against molecular oxygen. Mutational experiments for *At*64 and experiments in the presence and absence of 8-HDF for *Xl*64 and *Cr*aCRY showed that the local structure of the secondary pocket critically affected the kinetic stability of FADH^−^. Contrary to the well-known function of 8-HDF as an energy-transferring antenna chromophore, our observation that 8-HDF enhanced the tolerance of the active FADH^−^ state against reoxidation implies an undefined role for 8-HDF in maintaining the readiness of photolyases for their repair reaction. Although no antenna chromophore has been reported for *At*64, our study also suggested that the extended loop around the secondary pocket in *At*64 would be evolutionarily acquired to increase the kinetic stability of FADH^−^ and compensate for the lack of the antenna chromophore.

In addition to its ability to repair (6–4)PPs, *Cr*aCRY has garnered significant interest because of its CRY function to transduce light signals into other proteins using blue light. In addition, *Cr*aCRY is reportedly able to regulate the transcription of various genes under red light^26^. Indeed, some studies have suggested that *Cr*aCRY adopts the red-light-absorbing FADH^·^ state as its resting state^26,36,37^. However, a recent study showed that the conversion of FAD_ox_ into FADH^·^ turned on the interaction between *Cr*aCRY and a partner protein, ROC15 (Rhythm of Chloroplast 15), to synchronize the circadian clock with light^38^. Considering these paradoxical results, the resting and signaling states of FAD required for *Cr*aCRY to perform CRY function remain unclear. Our study provides beneficial clues to solve this problem. In the redox potential measurements of FAD, the X/XO reaction gradually lowered the redox potential of the system but did not demonstrate accumulation of the FADH^·^ state to detectable levels, indicating that the midpoint potential of the FAD_ox_/FADH^·^ redox couple should be significantly lower than that of the FADH^·^/FADH^−^ couple. Even if a low-potential environment is provided in *Chlamydomonas reinhardtii*, FADH^·^ would not be concentrated but would be immediately reduced to FADH^−^. Therefore, this discussion excludes the possibility that the FADH^·^ state is thermodynamically stabilized in *Chlamydomonas reinhardtii*.

Assuming that the FAD_ox_ state is the resting state in *Cr*aCRY from the above viewpoint of thermodynamics, FAD_ox_ needs to be photoreduced to FADH^·^ and/or FADH^−^ to express its functions as a photoreceptive CRY and a (6–4)PP-repairing protein. Because the dark reversion to FAD_ox_, which is conducted by the reoxidation by molecular oxygen, turns off the CRY function, the reoxidation kinetics of the potential signaling states (FADH^·^ and/or FADH^−^) are essential for the photoreceptive function. Our kinetic studies showed that the binding of 8-HDF to *Cr*aCRY extended the half-life of the FADH^−^ state from 1.3 to 5.6 h at 15 °C. Furthermore, a previous study reported that 8-HDF increases the kinetic stability of FADH^·^ against molecular oxygen (by ~ 3.5-fold)^36^. These studies may suggest that the presence of 8-HDF unnecessarily prolongs the life of the signaling states and decelerates the dark recovery of the resting state. Therefore, the binding of 8-HDF to *Cr*aCRY could be disadvantageous for sensing light/dark conditions, although our study cannot exclude the possibility that the kinetically stabilized FADH^·^ state produced by photoreduction could act as a transient resting state to be further photoreduced to FADH^−^ for the photoreceptive functions with 8-HDF. On the other hand, the prolonged lifetime of FADH^−^ is clearly beneficial for executing DNA repair, as the FADH^−^ state is exclusively required for the enzymatic reaction. Therefore, our kinetic study suggests a role of 8-HDF in shifting the function of *Cr*aCRY from the light-dependent signaling to the (6–4)PP-repair.

In conclusion, our study suggests that the biological functions of *Cr*aCRY could be switched by the presence or absence of 8-HDF, that is, CRY functions could be mediated by the photoreduction of FAD_ox_ to FADH^·^ in the absence of 8-HDF and the PL function with 8-HDF. To perform the CRY and PL functions properly, *Cr*aCRY in reduced FAD states may adopt different overall structures in the presence and absence of 8-HDF. To support this hypothesis presented by this study, we will investigate the structural effects of 8-HDF on *Cr*aCRY in various redox states in the near future.

## Methods

### Plasmid construction

In this study, we produced recombinant *At*64, *At*64-ΔExLoop, *Xl*64, and *Cr*aCRY using the pET28a(+) plasmid system. *At*64- and *Xl*64-expressing plasmids were constructed, as shown in the previous study^39^. The plasmid producing the *Cr*aCRY protein, which lacks the C-terminal tail (497–595)^27^ was kindly provided by Prof. Lars-Oliver Essen.

For the construction of the plasmid encoding *At*64-ΔExLoop, we separately amplified two fragments, excluding the extended loop (from Ser46 to Pro53) using the following sets of PCR primers: d(CCGCGCGGCAGCCATATGGCTACTGGATCCGGT) and d(GGCGCGAGACGAACCCTCCATATAATGCGGGTCGA) for 5’ upstream of the deletion site; d(CCGCATTATATGGAGGGTTCGTCTCGCGCC) and d(GTGGTGGTGCTCGAGCTATTTGAGTTTTGGTCGTTG) for 3’ downstream of the deletion site. The two fragments were integrated into the pET28a(+) vector linearized by *Nde*I and *Xho*I using an In-fusion HD Cloning Kit (Takara).

All plasmids were sequenced prior to the protein production.

### Protein purification

To prepare *At*64 and *Xl*64, *E. coli* C41 (DE3)/pLysS (Lucigen) cells were transformed with the pET-28a(+) plasmid encoding *At*64 or *Xl*64 gene, and protein production and purification were performed as previously described^31,39^. Preparation of *At*64-ΔExLoop was performed using the same procedure as that used for *At*64.

For *Cr*aCRY preparation, we followed a previously reported protocol^26^ except for some modifications. *E. coli* BL21 (DE3) cells were transformed with the pET-28a(+) plasmid expressing *Cr*aCRY and grown in a 2 L of Terrific Broth medium containing kanamycin (20 µg mL^−1^) in a 5 L flask with baffles at 37 °C. When OD_600_ reached 0.2, the culture was cooled and further grown at 18 °C. At an OD_600_ of 0.5, the protein production was induced using isopropyl-β-D-thiogalactopyranoside (10 µM final concentration), and the cells were further incubated at 18 °C for 24 h. After harvesting the cells, the pellets were frozen in liquid nitrogen and thawed on ice. The cells were resuspended in 30 mL of a loading buffer (50 mM phosphate, 100 mM NaCl, 20% glycerol, pH 7.8) containing 65 mg of lysozyme and lysed by sonication. After centrifugation of the lysate, the supernatant was loaded onto an open column filled with TALON Metal Affinity Resin (Takara) equilibrated with the loading buffer. After washing out the non-specifically bound proteins with five-column volumes of the loading buffer followed by five-column volumes of a wash buffer (50 mM phosphate, 100 mM NaCl, 25 mM imidazole, 20% glycerol, pH 7.8), the His-tagged protein was eluted with an elution buffer (50 mM phosphate, 100 mM NaCl, 250 mM imidazole, 20% glycerol, pH 7.8). The green eluate was further purified using a HiTrap Heparin HP column (Cytiva) with a linear gradient of 100–500 mM NaCl in a buffer containing 50 mM phosphate (pH 7.8) and 20% glycerol.

All purified proteins were analyzed by 10% SDS-PAGE, and their concentrations were determined based on the absorbance at 450 nm using the molar extinction coefficient of FAD in a (6–4) PL as 11 200 L mol^−1^ cm^−1^.

### Midpoint potential measurements by xanthine/xanthine oxidase method

The midpoint potentials of FAD_ox_/FADH^−^ (*E*_m,FAD_) in (6–4) PLs and *Cr*aCRY were measured using a method based on slow electron supply from the oxidation of xanthine by xanthine oxidase^29^. Briefly, the buffer in the protein solution was exchanged to a redox buffer (20 mM phosphate, 500 mM NaCl, 10% glycerol, pH 7.0) with a Micro Bio-Spin 6 column (BIO-RAD) and mixed with Safranin T solution. The volume of the protein and Safranin T was determined to ensure an absorbance of > 0.2 at 450 nm and 510 nm (typically, the final concentrations of the protein and Safranin T were approximately 30 and 10 µM, respectively). The sample was transferred into a 10 × 2 × 8 mm (length × width × height) inner volume quartz cuvette (Starna, 16.160-F/4/Q/10 GL 14/2/Z15) and degassed through the cap with rubber septa. In an anaerobic glove box (< 1 ppm of O_2_ level), the redox mediator methylviologen (30 µM final conc.), xanthine (300 µM final conc.), glucose (10 mM final conc.), 25 µg glucose oxidase (0.78 µM final conc.), and 50 µg catalase (1 µM final conc.) were added to the cuvette. After incubation for 15 min to decompose the remaining oxygen using glucose, glucose oxidase, and catalase, xanthine oxidase (94 nM final conc.) was added to initiate electron supply to the system. The absorption changes at two selected wavelengths (two of 450 nm, 510 nm, and 635 mm) were recorded every 10 s for 1 h or 3 h using a V-730 spectrometer (JASCO). To check the structural integrity and reaction progress, UV/vis spectra were recorded before and after the measurement using a Lambda 35 UV–vis spectrometer (PerkinElmer). The obtained data were analyzed using Eq. 1. Three independent experiments were conducted for each sample.

### Monitoring the reoxidation kinetics by UV/vis absorption spectroscopy

To monitor reoxidation kinetics, FAD in a protein was photoreduced under anaerobic conditions as previously described^39^. An anaerobic sample containing ~ 5 µM protein and 5 mM L-cysteine in a reaction buffer (20 mM phosphate, 500 mM NaCl, 10% glycerol, pH 7.5), which was prepared in the cuvette used for the midpoint potential measurement, was illuminated with continuous light (430–800 nm) from a MAX-150 xenon lamp (Asahi Spectra) through a 10 × 8 mm window of the cuvette on ice. After confirming the reduction by UV/vis absorption measurements, the reduced sample was exposed to air by removing the lid of the cuvette for the reoxidation of FAD. The sample was placed in the V-750 spectrometer (JASCO), and the temperature of the sample holder was maintained at 15 °C. The progress of the reoxidation was monitored every 10 min for 2 h by measuring the UV/vis spectra.

To prepare 8-HDF-binding proteins, 20 µM protein was incubated with 100 µM 8-HDF in 70 µL buffer (50 mM Tris–HCl, 5% glycerol, 500 mM NaCl) for 30 min on ice. After the sample was applied to a Micro Bio-Spin 6 column (Bio-Rad) equilibrated with the reaction buffer, the eluate was used for sample preparation, as mentioned above.

### Supplementary Information

Supplementary Information.

## Data Availability

The datasets generated during and/or analyzed during the current study are available from the corresponding author on reasonable request.

## References

[1] You YH (2001). Cyclobutane pyrimidine dimers are responsible for the vast majority of mutations induced by UVB irradiation in mammalian cells. J. Biol. Chem..

[2] Lo HL (2005). Differential biologic effects of CPD and 6–4PP UV-induced DNA damage on the induction of apoptosis and cell-cycle arrest. BMC Cancer.

[3] Sancar A (2003). Structure and function of DNA photolyase and cryptochrome blue-light photoreceptors. Chem. Rev..

[4] Liu Z, Wang L, Zhong D (2015). Dynamics and mechanisms of DNA repair by photolyase. Phys. Chem. Chem. Phys..

[5] Yamamoto J, Plaza P, Brettel K (2017). Repair of (6–4) lesions in DNA by (6–4) photolyase: 20 years of quest for the photoreaction mechanism. Photochem. Photobiol..

[6] Wang J, Du X, Pan W, Wang X, Wu W (2015). Photoactivation of the cryptochrome/photolyase superfamily. J. Photochem. Photobiol. C: Photochem. Rev..

[7] Tan C (2014). Direct determination of resonance energy transfer in photolyase: structural alignment for the functional state. J. Phys. Chem. A.

[8] Lipman RS, Jorns MS (1992). Direct evidence for singlet-singlet energy transfer in *Escherichia coli* DNA photolyase. Biochemistry.

[9] Selby CP, Sancar A (2012). The second chromophore in *Drosophila* photolyase/cryptochrome family photoreceptors. Biochemistry.

[10] Glas AF (2009). The archaeal cofactor F0 is a light-harvesting antenna chromophore in eukaryotes. Proc. Natl. Acad. Sci. USA.

[11] Chen S (2022). Identification and characterization of a prokaryotic 6–4 photolyase from *Synechococcus elongatus* with a deazariboflavin antenna chromophore. Nucleic Acids Res..

[12] Morimoto A (2021). Key interactions with deazariboflavin cofactor for light-driven energy transfer in *Xenopus* (6–4) photolyase. Photochem. Photobiol. Sci..

[13] Chaves I (2011). The cryptochromes: Blue light photoreceptors in plants and animals. Annu. Rev. Plant. Biol..

[14] Guo H, Yang T, Mockler TC, Lin C (1998). Regulation of flowering time by *Arabidopsis* photoreceptors. Science.

[15] Xu L (2008). Active site of *Escherichia coli* DNA photolyase: Asn378 is crucial both for stabilizing the neutral flavin radical cofactor and for DNA repair. Biochemistry.

[16] Wang X, Wang Q, Nguyen P, Lin C (2014). Cryptochrome-mediated light responses in plants. Enzymes.

[17] Toth R (2001). Circadian clock-regulated expression of phytochrome and cryptochrome genes in *Arabidopsis*. Plant Physiol..

[18] Emery P, So WV, Kaneko M, Hall JC, Rosbash M (1998). CRY, a *Drosophila* clock and light-regulated cryptochrome, is a major contributor to circadian rhythm resetting and photosensitivity. Cell.

[19] Banerjee R (2007). The signaling state of *Arabidopsis* cryptochrome 2 contains flavin semiquinone. J. Biol. Chem..

[20] Vaidya AT (2013). Flavin reduction activates *Drosophila* cryptochrome. Proc. Natl. Acad. Sci. USA.

[21] Balland V, Byrdin M, Eker AP, Ahmad M, Brettel K (2009). What makes the difference between a cryptochrome and DNA photolyase? A spectroelectrochemical comparison of the flavin redox transitions. J. Am. Chem. Soc..

[22] Langenbacher T, Immeln D, Dick B, Kottke T (2009). Microsecond light-induced proton transfer to flavin in the blue light sensor plant cryptochrome. J. Am. Chem. Soc..

[23] Paulus B (2015). Spectroscopic characterization of radicals and radical pairs in fruit fly cryptochrome–protonated and nonprotonated flavin radical-states. FEBS J..

[24] Damiani MJ, Nostedt JJ, O'Neill MA (2011). Impact of the N5-proximal Asn on the thermodynamic and kinetic stability of the semiquinone radical in photolyase. J. Biol. Chem..

[25] Maestre-Reyna M (2022). Serial crystallography captures dynamic control of sequential electron and proton transfer events in a flavoenzyme. Nat. Chem..

[26] Beel B (2012). A flavin binding cryptochrome photoreceptor responds to both blue and red light in *Chlamydomonas reinhardtii*. Plant Cell.

[27] Franz S (2018). Structure of the bifunctional cryptochrome aCRY from *Chlamydomonas reinhardtii*. Nucleic Acids Res..

[28] Oldemeyer S (2016). Essential role of an unusually long-lived tyrosyl radical in the response to red light of the animal-like cryptochrome aCRY. J. Biol. Chem..

[29] Maklashina E, Cecchini G (2020). Determination of flavin potential in proteins by xanthine/xanthine oxidase method. Bio. Protoc..

[30] Kavakli IH, Sancar A (2004). Analysis of the role of intraprotein electron transfer in photoreactivation by DNA photolyase in vivo. Biochemistry.

[31] Hosokawa Y, Sato R, Iwai S, Yamamoto J (2019). Implications of a water molecule for photoactivation of plant (6–4) photolyase. J. Phys. Chem. B.

[32] Hitomi K (2009). Functional motifs in the (6–4) photolyase crystal structure make a comparative framework for DNA repair photolyases and clock cryptochromes. Proc. Natl. Acad. Sci. USA.

[33] Massey V (1994). Activation of molecular oxygen by flavins and flavoproteins. J. Biol. Chem..

[34] Müller P, Ahmad M (2011). Light-activated cryptochrome reacts with molecular oxygen to form a flavin-superoxide radical pair consistent with magnetoreception. J. Biol. Chem..

[35] Rosensweig C (2018). An evolutionary hotspot defines functional differences between CRYPTOCHROMES. Nat. Commun..

[36] Oldemeyer S, Haddad AZ, Fleming GR (2020). Interconnection of the antenna pigment 8-HDF and flavin facilitates red-light reception in a bifunctional animal-like cryptochrome. Biochemistry.

[37] Zou Y (2017). An Animal-like cryptochrome controls the *Chlamydomonas* sexual cycle. Plant Physiol..

[38] Li P (2022). Direct experimental observation of blue-light-induced conformational change and intermolecular interactions of cryptochrome. Commun. Biol..

[39] Hosokawa Y, Müller P, Kitoh-Nishioka H, Iwai S, Yamamoto J (2022). Limited solvation of an electron donating tryptophan stabilizes a photoinduced charge-separated state in plant (6–4) photolyase. Sci. Rep..

