# Supplementary material for: A deazariboflavin chromophore kinetically stabilizes reduced FAD state in a bifunctional cryptochrome
Source: Sci Rep. 2023 Oct 4;13:16682. doi: 10.1038/s41598-023-43930-0 (PMC10551024; doi:10.1038/s41598-023-43930-0)
Supplement: Supplementary file 1 — Supplementary Information. [file 41598_2023_43930_MOESM1_ESM.pdf]

Supplementary Information

A deazariboflavin chromophore kinetically stabilizes reduced FAD state in a bifunctional cryptochrome

Yuhei Hosokawa, Hiroyoshi Morita, Mai Nakamura, and Junpei Yamamoto*

Graduate School of Engineering Science, Osaka University, 1-3 Machikaneyama, Toyonaka, Osaka 560-8531, Japan.

*To whom correspondence should be addressed.

Junpei Yamamoto – Tel. and Fax: +81 6 6850 6240; E-mail: yamamoto.junpei.es@osaka-u.ac.jp

Supplementary Methods

Theoretical part of the midpoint potential measurement of FAD by xanthine/xanthine oxidase method

When the oxidation of xanthine (X) by xanthine oxidase (XO) supplies electrons to FAD and a reference dye without accumulating their one-electron reduced forms, the Nernst equation is applied to each redox active compound, as follows[1].

$$E_{\mathrm{FAD}} = E_{\mathrm{m,FAD}} + \frac{RT}{2F}\ln\frac{[\mathrm{FAD_{ox}}]_t}{[\mathrm{FADH^-}]_t} \quad \text{for FAD in protein} \qquad (\mathrm{S1})$$

$$E_{\mathrm{D}} = E_{\mathrm{m,D}} + \frac{RT}{2F}\ln\frac{[\mathrm{D_{ox}}]_t}{[\mathrm{D_{red}}]_t} \quad \text{for a reference dye} \qquad (\mathrm{S2})$$

In these equations, reduction potentials of FAD and the reference dye represented as E_{FAD} and E_{D}, respectively, are related to their concentration ratios between oxidized and two-electron reduced states ($[\mathrm{FAD_{ox}}]_t$ and $[\mathrm{FADH^-}]_t$ for FAD, $[\mathrm{D_{ox}}]_t$ and $[\mathrm{D_{red}}]_t$ for the dye, where t indicates the time at which the absorbance is measured) and their midpoint potentials ($E_{\mathrm{m,FAD}}$ and $E_{\mathrm{m,D}}$). R is the gas constant (8.314 J K^{-1} mol^{-1}), F is the Faraday constant (96,485 C mol^{-1}), and T is the absolute temperature (298 K in the experiment). Supposing that their midpoint potentials are similar to each other (in a range of 30 mV), the reduction potentials should be equal to each other ($E_{\mathrm{FAD}} = E_{\mathrm{D}}$), and the following equation holds.

$$E_{m,FAD} + \frac{RT}{2F}\ln\frac{[\mathrm{FAD_{ox}}]_t}{[\mathrm{FADH^-}]_t} = E_{m,D} + \frac{RT}{2F}\ln\frac{[\mathrm{D_{ox}}]_t}{[\mathrm{D_{red}}]_t} \qquad (S3)$$

By substituting constant values into Equation S3, Equation S4 is obtained for the midpoint potential analysis of the $\mathrm{FAD_{ox}/FADH^-}$ couple in millivolts.

$$12.8\ln\frac{[\mathrm{D_{ox}}]_t}{[\mathrm{D_{red}}]_t} = 12.8\ln\frac{[\mathrm{FAD_{ox}}]_t}{[\mathrm{FADH^-}]_t} + E_{\mathrm{m,FAD}} - E_{\mathrm{m,D}} \qquad (S4)$$

Kinetic model of the FAD reoxidation

The reoxidation of reduced FAD species in (6–4) PLs and *Cr*aCRY can be described by Supplementary Scheme S1[2].

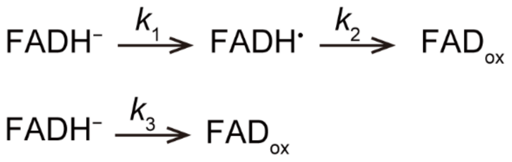

Supplementary Scheme S1. The whole reoxidation scheme in (6–4) PLs and *Cr*aCRY

The rate constants of k_1, k_2, and k_3 in Supplementary Scheme S1 are related to the concentrations of redox forms of FAD as follows.

$$\frac{d[\mathrm{FADH}^-]_t}{dt} = -k_1[\mathrm{FADH}^-]_t - k_3[\mathrm{FADH}^-]_t \qquad (S5)$$

$$\frac{d[\mathrm{FADH}^\bullet]_t}{dt} = k_1[\mathrm{FADH}^-]_t - k_2[\mathrm{FADH}^\bullet]_t \qquad (S6)$$

$$\frac{d[\mathrm{FAD_{ox}}]_t}{dt} = k_2[\mathrm{FADH}^\bullet]_t + k_3[\mathrm{FADH}^-]_t \qquad (S7)$$

By solving Equations S5 and S6, we could describe the time development of the concentrations of FADH^- and $\mathrm{FADH}^\bullet$ ($\Delta[\mathrm{FADH}^-]$ and $\Delta[\mathrm{FADH}^\bullet]$, in which Δ means the changes in the concentration of a reactant over a specific time interval), as shown in Equations S8 and S9.

$$\Delta[\mathrm{FADH}^-] = [\mathrm{FADH}^-]_0[exp\{-(k_1+k_3)t\} - 1] \qquad (S8)$$

$$\Delta[\mathrm{FADH}^\bullet] = [\mathrm{FADH}^-]_0\frac{k_1}{k_2-k_1-k_3}[exp\{-(k_1+k_3)t\} - exp\,(-k_2t)] \\ +[\mathrm{FADH}^\bullet]_0\{exp(-k_2t) - 1\} \qquad (S9)$$

Considering that the total concentration of FAD is the summation of the concentrations of FADH^-, $\mathrm{FADH}^\bullet$, and $\mathrm{FAD_{ox}}$, $\Delta[\mathrm{FAD_{ox}}]$ should be described by Equation S10.

$$\Delta[\mathrm{FAD_{ox}}] = -[\mathrm{FADH}^-]_0\frac{k_2-k_3}{k_2-k_1-k_3}[exp\{-(k_1+k_3)t\} - 1] \\ +[\mathrm{FADH}^-]_0\frac{k_1}{k_2-k_1-k_3}\{exp(-k_2t) - 1\} - [\mathrm{FADH}^\bullet]_0\{exp(-k_2t) - 1\} \qquad (S10)$$

However, the k_2 value in our tested proteins is much smaller than k_1 and/or k_3 as discussed in *Results*. The $k_2 \ll k_1 + k_3$ approximation can be applied to Equations S9 and S10.

$$\Delta[\mathrm{FADH}^\bullet] = -[\mathrm{FADH}^-]_0\frac{k_1}{k_1+k_3}[exp\{-(k_1+k_3)t\} - 1] \qquad (S11)$$

$$\Delta[\mathrm{FAD_{ox}}] = -[\mathrm{FADH^-}]_0 \frac{k_3}{k_1 + k_3}[exp\{-(k_1 + k_3)t\} - 1] \quad (S12)$$

For *At*64, *At*64-ΔExLoop, and *Cr*aCRY-HDF, the absorption changes unique to the reoxidation were observed to be fitted not with a single exponential component but with a line because of their slow reoxidation kinetics. When we analyzed these data, we used Equations S13, S14, and S15, which were derived from the linear approximation of Equations S8, S11, and S12, respectively.

$$\Delta[\mathrm{FADH^-}] \approx -(k_1 + k_3)[\mathrm{FADH^-}]_0 t \quad (S13)$$

$$\Delta[\mathrm{FADH^\bullet}] \approx k_1[\mathrm{FADH^-}]_0 t \quad (S14)$$

$$\Delta[\mathrm{FAD_{ox}}] \approx k_3[\mathrm{FADH^-}]_0 t \quad (S15)$$

Based on the obtained absorption changes, the Beer-Lambert law ($A = \varepsilon cl$) and Equations S8, and S11–15, we calculated the rate constants of k_1 and k_3 and the time-dependent concentrations of $[\mathrm{FADH^-}]_t$, $[\mathrm{FADH^\bullet}]_t$, and $[\mathrm{FAD_{ox}}]_t$. The values of molar extinction coefficients used for the analyses are shown in Supplementary Table 1.

Supplementary Table and Figures

Supplementary Table 1 The values of molar extinction coefficients of FAD_{ox}, $FADH^{\bullet}$, and $FADH^{-}$

Wavelength (nm)	ε^{a} (L mol^{-1} cm^{-1})		
	FAD_{ox}	$FADH^{\bullet}$	$FADH^{-}$
450	11204	3833	1634
635	~0	5033	~0

[a] Ref. 3 in Supplementary References

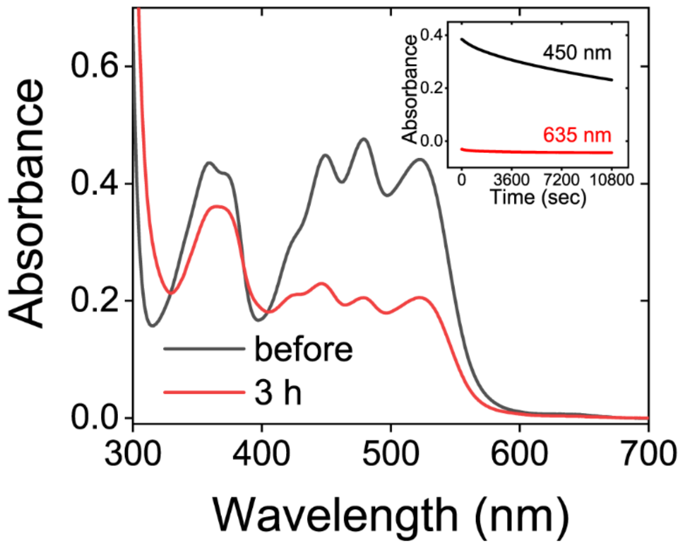

Supplementary Figure 1. No accumulation of the one-electron reduced $FADH^{\bullet}$ state during the simultaneous reduction of FAD in *At*64 and Safranin T. The inset shows that the absorption unique to $FADH^{\bullet}$ at 635 nm was not increasing even if the FAD_{ox} state was gradually reduced as shown in the decreasing absorption at 450 nm. Comparison of UV/vis spectra measured before and 3 h after the reaction indicated that large portions of FAD and Safranin T were converted to their two-electron reduced states without concentrating $FADH^{\bullet}$.

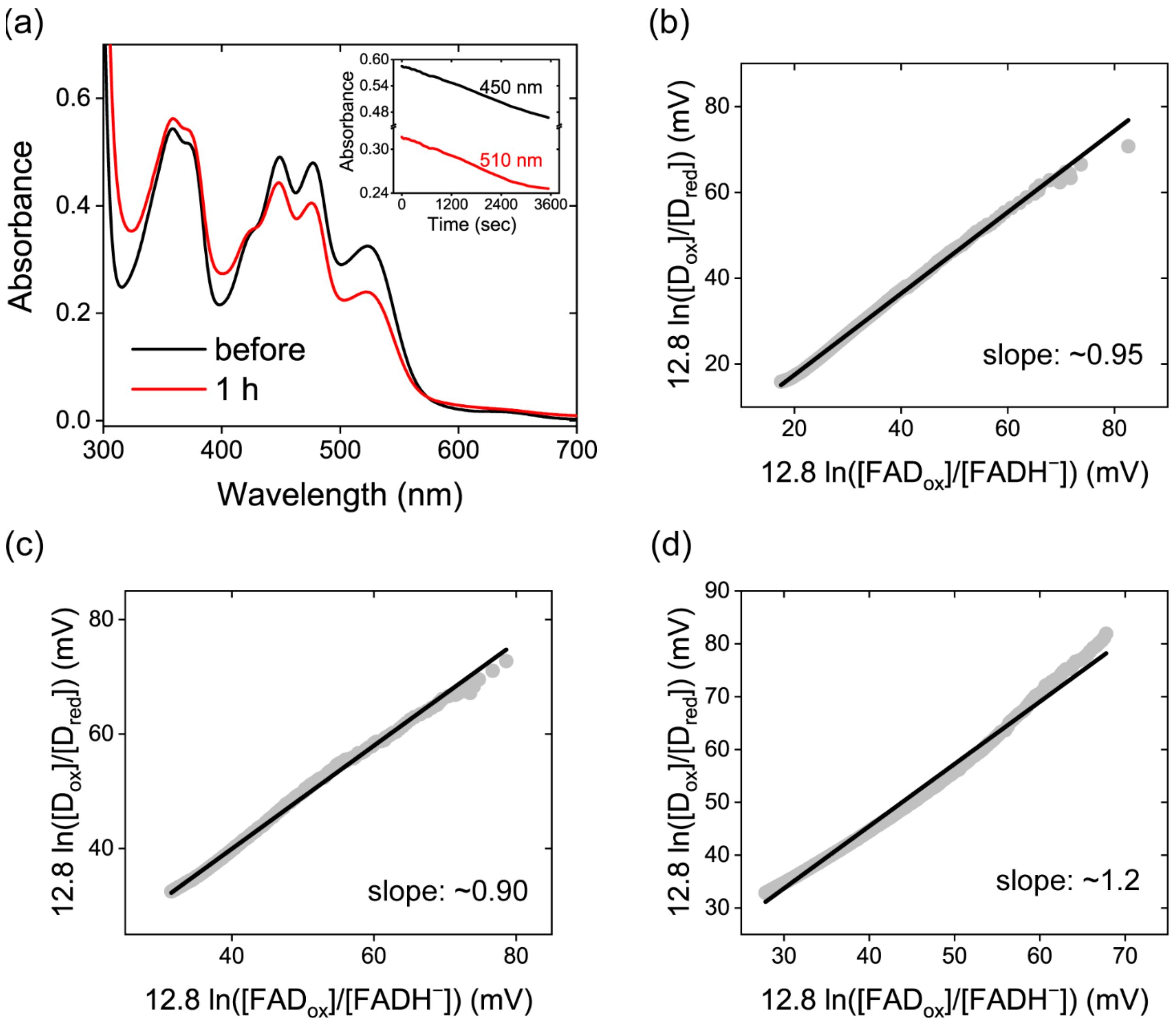

Supplementary Figure 2. Midpoint potential measurements of FAD in *At*64 by the xanthine/xanthine oxidase method. (a) The representative UV/vis absorption spectra measured before and 1 h after the reaction indicated that FAD and Safranin T were gradually converted to their two-electron reduced states without concentrating FADH$^{\bullet}$. The insets show the decay of the absorption at 450 nm and 510 nm unique to the fully oxidized FAD and Safranin T forms, respectively, during the measurement time of 1 h. (b), (c), (d) Three independent experimental data were analyzed by the plots (12.8 ln ([FAD$_{ox}$]$_t$/[FADH$^-$]$_t$) vs. 12.8 ln ([D$_{ox}$]$_t$/[D$_{red}$]$_t$)). The plots were approximated by a line for each experiment. The slope values of the lines described in the figures were close to 1, validating the analyses.

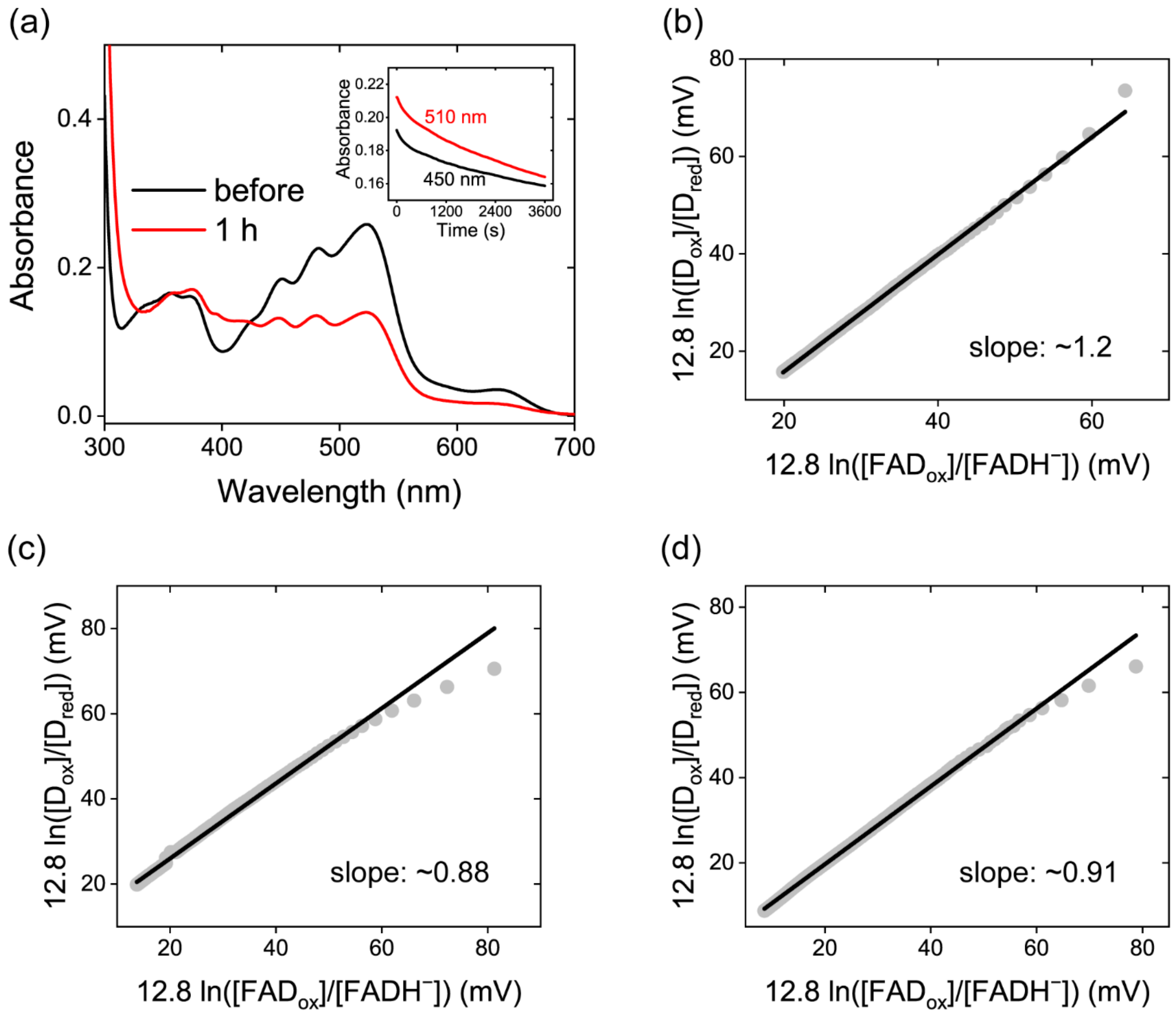

Supplementary Figure 3. Midpoint potential measurements of FAD in *Xl*64 by the xanthine/xanthine oxidase method. (a) The representative UV/vis absorption spectra measured before and 1 h after the reaction indicated that FAD and Safranin T were gradually converted to their two-electron reduced states without concentrating $FADH^{\bullet}$. The insets show the decay of the absorption at 450 nm and 510 nm unique to the fully oxidized FAD and Safranin T forms, respectively, during the measurement time of 1 h. (b), (c), (d) Three independent experimental data were analyzed by the plots (12.8 ln ($[FAD_{ox}]_t/[FADH^-]_t$) vs. 12.8 ln ($[D_{ox}]_t/[D_{red}]_t$)). The plots were approximated by a line for each experiment. The slope values of the lines described in the figures were close to 1, validating the analyses.

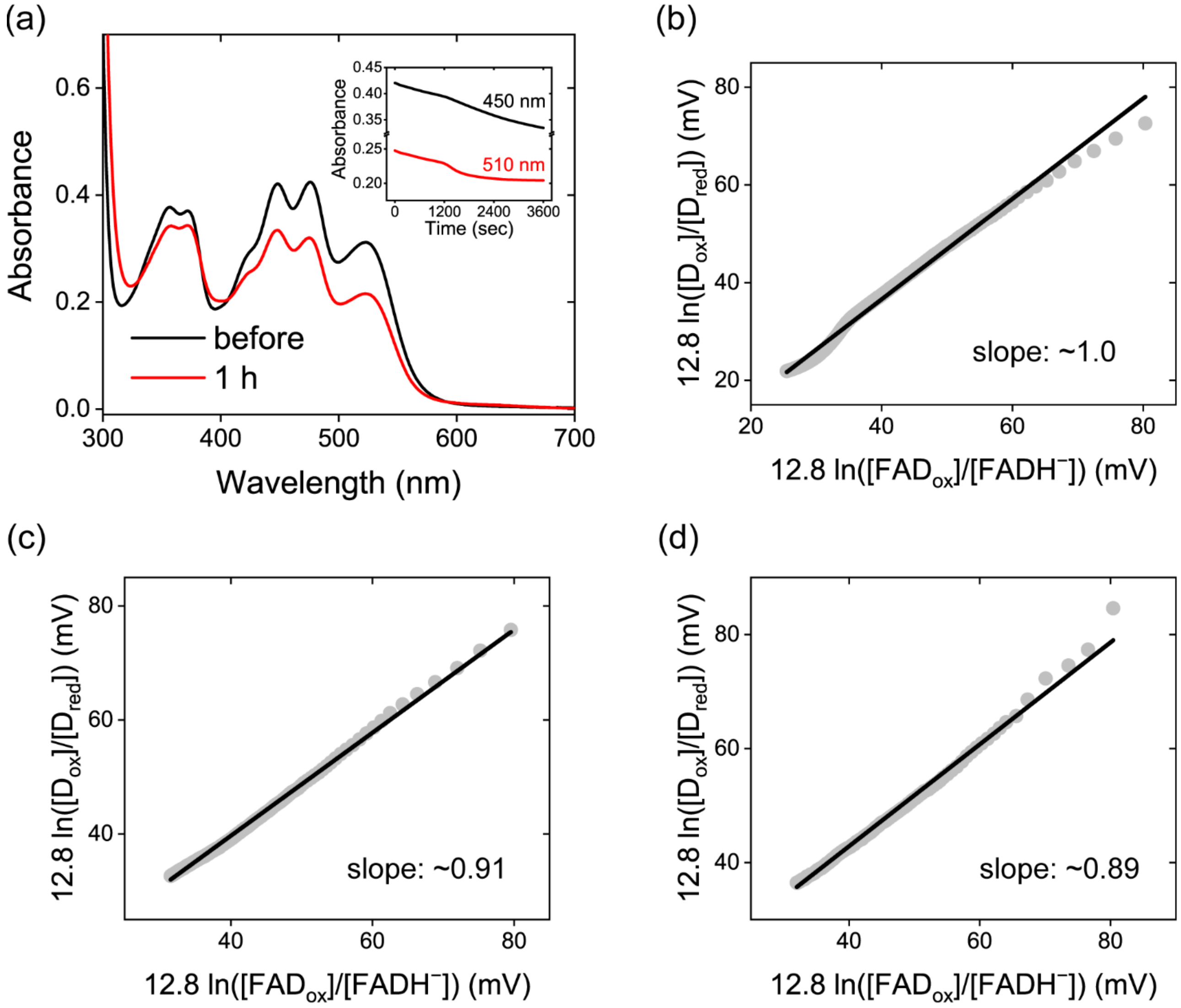

Supplementary Figure 4. Midpoint potential measurements of FAD in *Cr*aCRY by the xanthine/xanthine oxidase method. (a) The representative UV/vis absorption spectra measured before and 1 h after the reaction indicated that FAD and Safranin T were gradually converted to their two-electron reduced states without concentrating $FADH^{•}$. The insets show the decay of the absorption at 450 nm and 510 nm unique to the fully oxidized FAD and Safranin T forms, respectively, during the measurement time of 1 h. (b), (c), (d) Three independent experimental data were analyzed by the plots (12.8 ln ($[FAD_{ox}]_t/[FADH^-]_t$) vs. 12.8 ln ($[D_{ox}]_t/[D_{red}]_t$)). The plots were approximated by a line for each experiment. The slope values of the lines described in the figures were close to 1, validating the analyses.

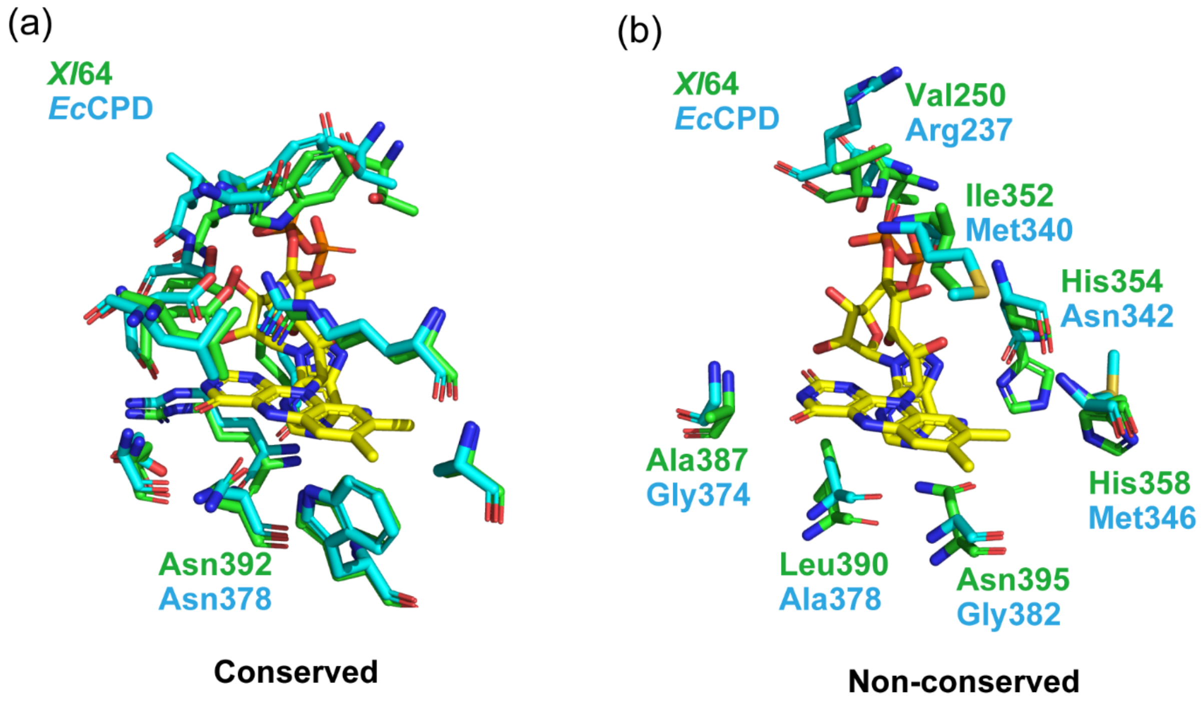

Supplementary Figure 5. Structural comparison of the FAD binding site within 4 Å of FAD between (6–4) PL from *Xenopus laevis* (*Xl*64) and CPD PL from *Escherichia coli* (*Ec*CPD). The used structures of *Xl*64 (in green) and *Ec*CPD (in cyan) were the model generated previously[4] and the crystal structure[5] (PDB ID: 1DNP), respectively. FAD is shown in yellow. (a) The conserved residues between *Xl*64 and *Ec*CPD are shown. The Asn residue next to the N5 atom of isoalloxazine in FAD is well known as an essential factor to control the stability of redox states of FAD and is conserved between *Xl*64 and *Ec*CPD (Asn392 for *Xl*64; Asn378 for *Ec*CPD). (b) The non-conserved residues between *Xl*64 and *Ec*CPD are shown. There are considerable differences in the active site between *Xl*64 and *Ec*CPD depending on the different substrates subjected to *Xl*64 and *Ec*CPD.

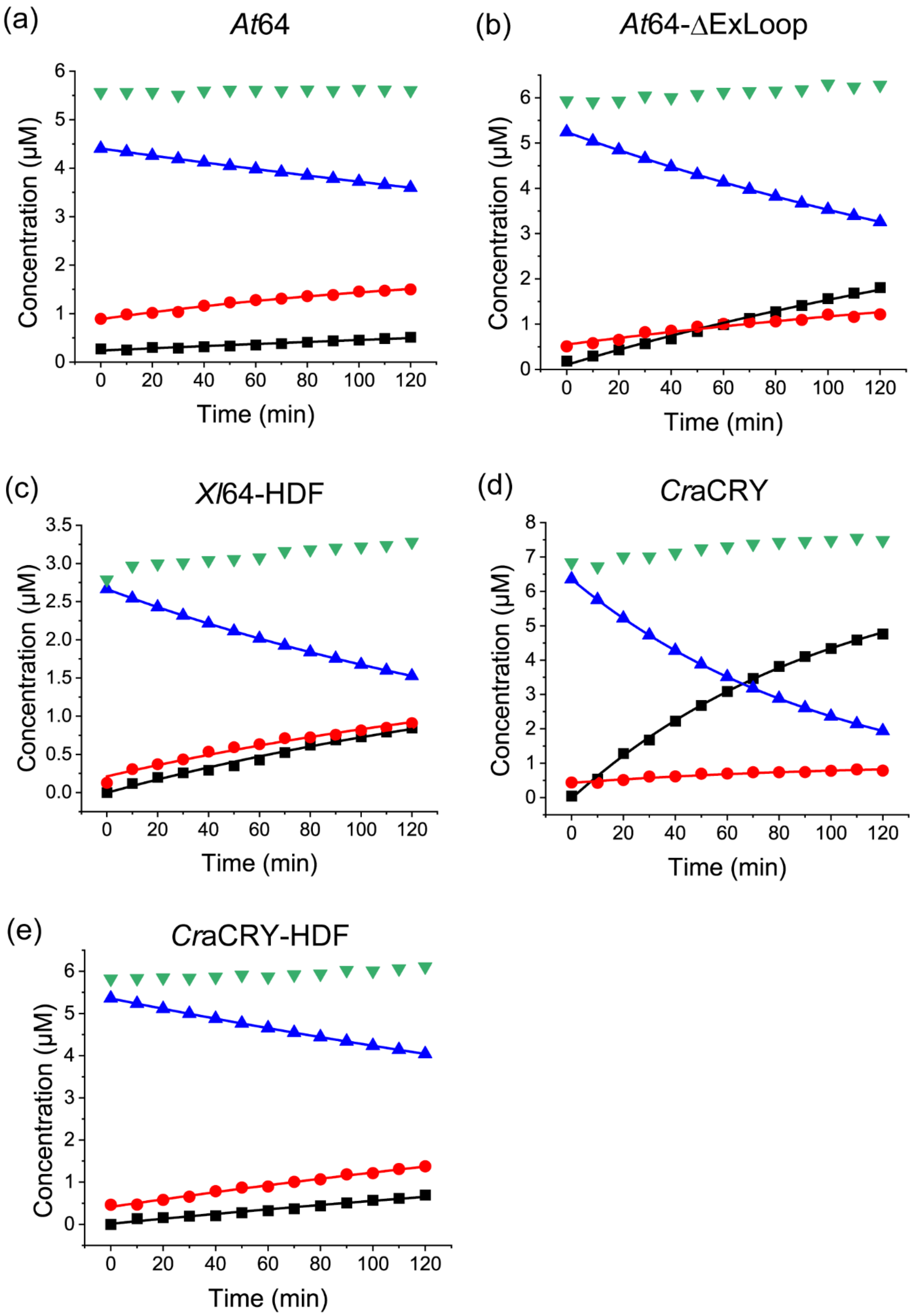

Supplementary Figure 6. The time-dependent concentrations of FAD_{ox} (black), $FADH^{\bullet}$ (red), and $FADH^{-}$ (blue) upon oxidation of the photoreduced (a) *At*64, (b) *At*64-ΔExLoop, (c) *Xl*64-HDF, (d) *Cr*aCRY, and (e) *Cr*aCRY-HDF samples. The points calculated from the corresponding UV/vis spectra are well fitted with a single exponential decay function with the rate constant of $k_1 + k_3$. The green symbols show total concentrations of FAD_{ox}, $FADH^{\bullet}$, and $FADH^{-}$. Here, we first obtained the $FADH^{\bullet}$ concentration based on the absorption at 635 nm, followed by

determination of the $k_1 + k_3$ value from the fitting of the FADH$^{\bullet}$ concentration with Eq. 11. The FADH^{-} concentration at each time point was extrapolated from the rate constant. Finally, the FAD_{ox} concentration was obtained using absorbance at 450 nm, after subtraction of the contributions of FADH$^{\bullet}$ and FADH^{-} at this wavelength. As the total FAD concentration remains nearly constant with a standard deviation of less than 5% for all the samples, the deconvolution of FAD concentrations was reasonably performed.

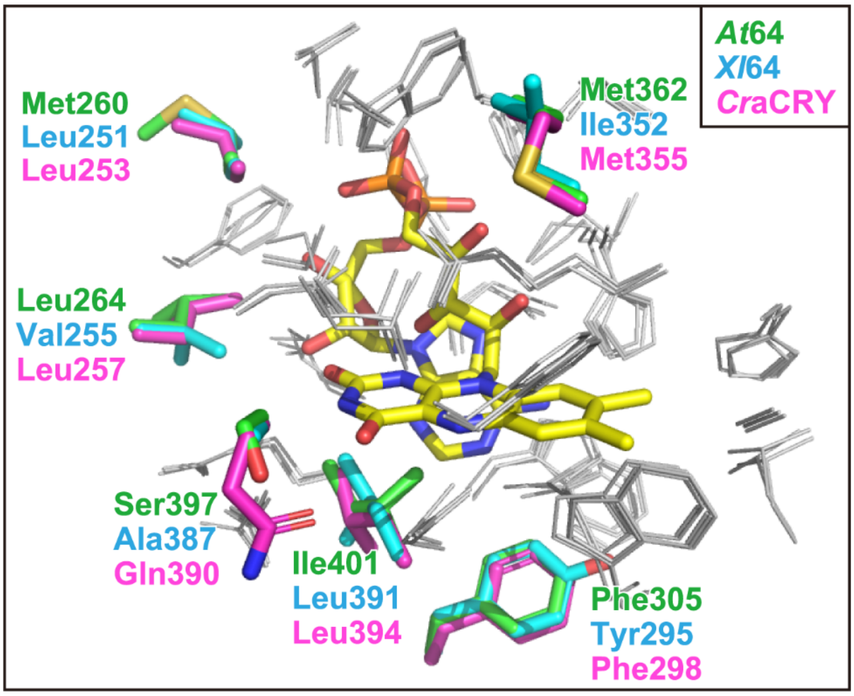

Supplementary Figure 7. Structural comparison of the FAD binding site within 5 Å of FAD among *Xl*64, *At*64, and *Cra*CRY. The used structure of *Xl*64 was the model generated previously[4], and the structures of *At*64 and *Cra*CRY were taken from the crystal structures[6,7] of PDB ID: 3FY4 and 6FN2, respectively. FAD is shown in yellow. The conserved residues among the proteins are shown in gray lines. The residues showing some varieties among the proteins are shown in sticks. The green, cyan, and magenta sticks correspond to the residues of *At*64, *Xl*64, and *Cra*CRY, respectively.

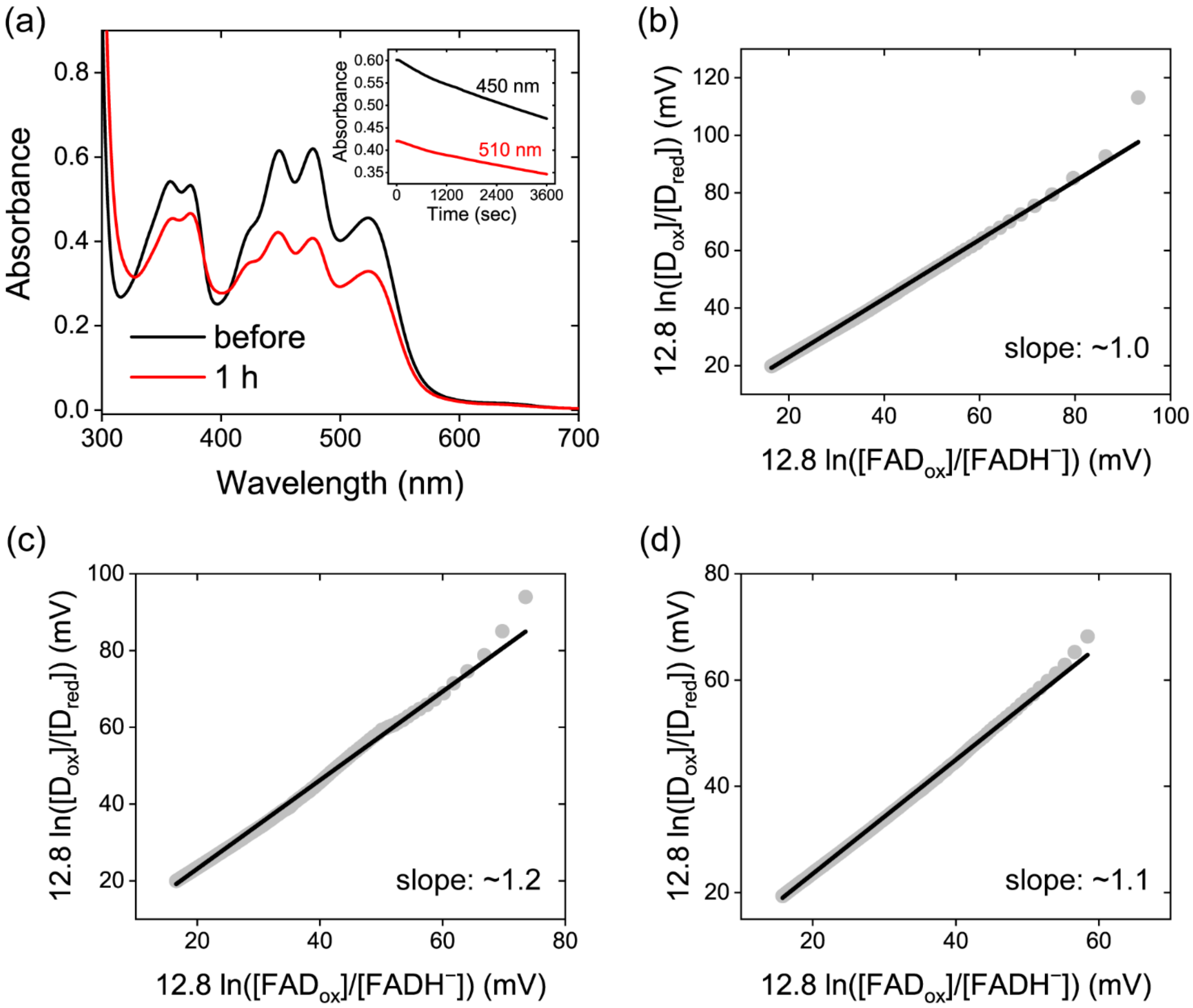

Supplementary Figure 8. Midpoint potential measurements of FAD in *At*64-ΔExLoop by the xanthine/xanthine oxidase method. (a) The representative UV/vis absorption spectra measured before and 1 h after the reaction indicated that FAD and Safranin T were gradually converted to their two-electron reduced states without concentrating FADH$^{\bullet}$. The insets show the decay of the absorption at 450 nm and 510 nm unique to the fully oxidized FAD and Safranin T forms, respectively, during the measurement time of 1 h. (b), (c), (d) Three independent experimental data were analyzed by the plots (12.8 ln ($[FAD_{ox}]_t/[FADH^-]_t$) vs. 12.8 ln ($[D_{ox}]_t/[D_{red}]_t$)). The plots were approximated by a line for each experiment. The slope values of the lines described in the figures were close to 1, validating the analyses.

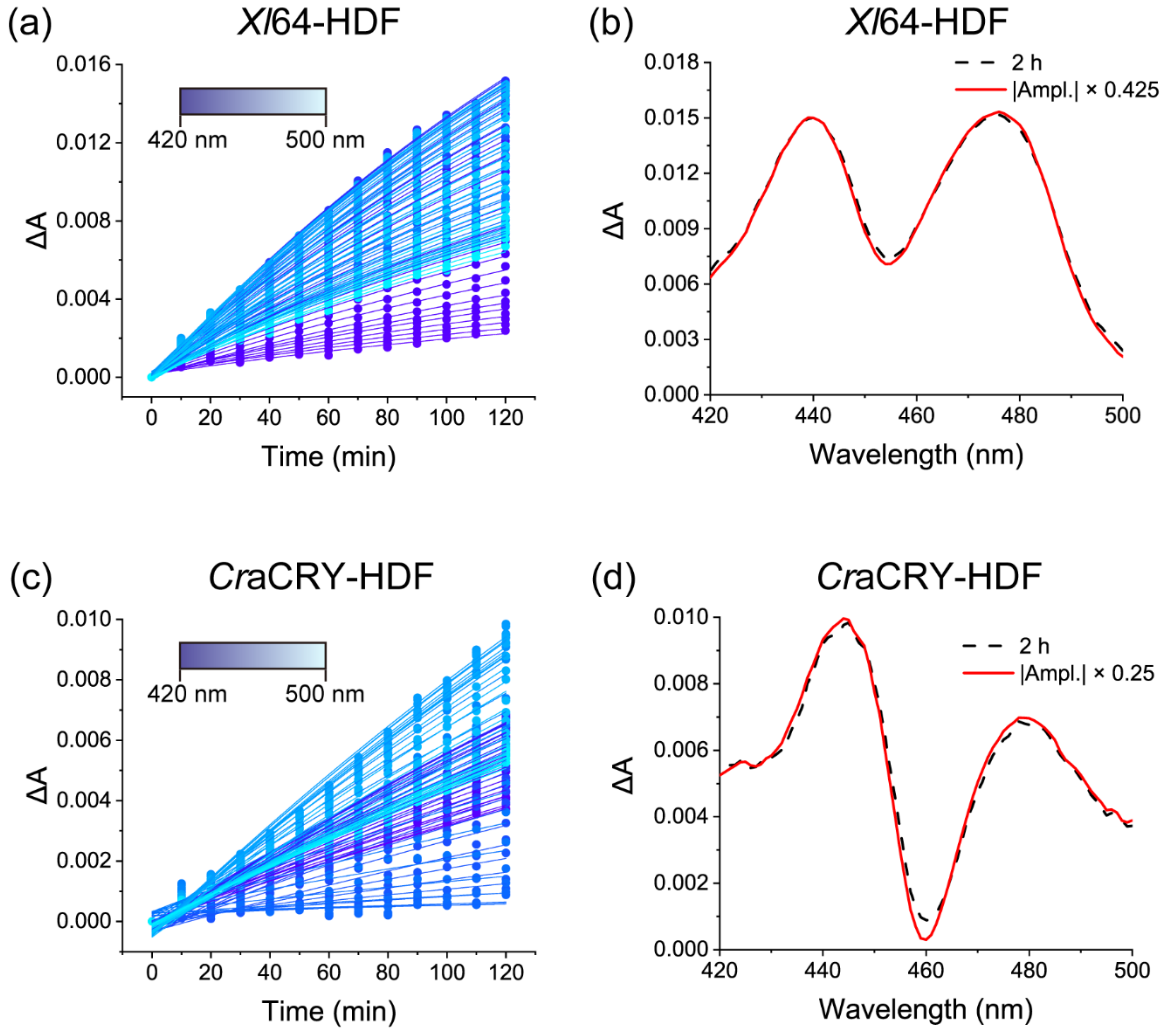

Supplementary Figure 9. Validation of kinetic analyses of *Xl*64-HDF and *Cra*CRY-HDF. The time-dependent absorption changes in the wavelength of 420–500 nm (dark–light blue) for (a) *Xl*64-HDF and (c) *Cra*CRY-HDF were globally fitted by single exponential functions using the obtained $k_1 + k_3$ values, and the coefficients of determination for (a) and (c) were 0.998 and 0.990, respectively. The amplitude spectra (red line) for (b) *Xl*64-HDF and (d) *Cra*CRY-HDF were scaled and compared with the individual difference spectrum upon the reoxidation for 2 h (broken line).

Suppelementary References

1 Maklashina, E. & Cecchini, G. Determination of Flavin Potential in Proteins by Xanthine/Xanthine Oxidase Method. *Bio. Protoc.* **10**, e3571. https://doi.org:10.21769/BioProtoc.3571 (2020).

2 Wen, B. *et al.* A single amino acid residue tunes the stability of the fully reduced flavin cofactor and photorepair activity in photolyases. *J. Biol. Chem.* **298**, 102188. https://doi.org:10.1016/j.jbc.2022.102188 (2022).

3 Yamamoto, J., Martin, R., Iwai, S., Plaza, P. & Brettel, K. Repair of the (6–4) photoproduct by DNA photolyase requires two photons. *Angew. Chem. Int. Ed.* **52**, 7432–7436 (2013).

4 Hosokawa, Y., Müller, P., Kitoh-Nishioka, H., Iwai, S. & Yamamoto, J. Limited solvation of an electron donating tryptophan stabilizes a photoinduced charge-separated state in plant (6–4) photolyase. *Sci. Rep.* **12**, 5084. https://doi.org:10.1038/s41598-022-08928-0 (2022).

5 Park, H. W., Kim, S. T., Sancar, A. & Deisenhofer, J. Crystal structure of DNA photolyase from *Escherichia coli*. *Science* **268**, 1866–1872 (1995).

6 Hitomi, K. *et al.* Functional motifs in the (6–4) photolyase crystal structure make a comparative framework for DNA repair photolyases and clock cryptochromes. *Proc. Natl. Acad. Sci. USA* **106**, 6962–6967 (2009).

7 Franz, S. *et al.* Structure of the bifunctional cryptochrome aCRY from *Chlamydomonas reinhardtii*. *Nucleic Acids Res.* **46**, 8010–8022 (2018).
